# Supplementary material for: Effect of a Simulated Match on Lower Limb Neuromuscular Performance in Youth Footballers—A Two Year Longitudinal Study
Source: Int J Environ Res Public Health. 2020 Nov 19;17(22):8579. doi: 10.3390/ijerph17228579 (PMC7699215; doi:10.3390/ijerph17228579)
Supplement: Supplementary file 1 [file ijerph-17-08579-s001.zip › ijerph-983107-supplementary-tables.docx]

Supplement

**Table S1.** Descriptive statistics for isokinetic parameters pre and post SAFT^90^, and magnitude-based inferences for the percent changes of the means in U16.

| **Parameter (N·m^-1^)** | **Pre-test (n=11)  Mean ± *SD*** | **Post-test (n=11)  Mean ± *SD*** | ***p*** | **Standardized**  **effect**  **size** | **Effect**  **Change in mean; ±90%CI** |
| --- | --- | --- | --- | --- | --- |
| **Concentric and eccentric action for hamstrings (PT; N·m^-1^)** | | | | |  |
| H Con 60 KL | 147.85 ± 20.04 | 148.85 ± 24.90 | 0.875 | <0.2 - trivial | - |
| H Con 180 KL | 175.33 ± 25.97 | 167.16 ± 26.45 | 0.182 | -0.29 - small | -4.7%; from -10.2% to 1.1 %, (possibly harmful [2, 31, 68]) |
| H Con 60 SL | 140.00 ± 34.6 | 130.00± 31.17 | 0.041* | -0.28 - small | -7.0%; from -12.1% to -1.6 %, (possibly harmful [0, 25, 75]) |
| H Con 180 SL | 158.88 ± 26.79 | 155.93 ± 22.51 | 0.410 | <0.2 - trivial | - |
| H Ecc 60 KL | 162.48 ± 32.67 | 148.84 ± 24.18 | 0.041* | -0.38 - small | -7.8%; from -14.6% to -0.5 %, (likely harmful [1, 18, 81]) |
| H Ecc 180 KL | 192.52 ± 28.25 | 186.70 ± 20.10 | 0.182 | <0.2 - trivial | - |
| H Ecc 60 SL | 147.04 ± 27.97 | 139.38 ± 25.69 | 0.019* | -0.27 - small | -5.1%; from -8.5% to -1.6 %, (possibly harmful [0, 27, 73]) |
| H Ecc 180 SL | 182.84 ± 21.19 | 185.83± 23.90 | 0.657 | <0.2 - trivial | - |
| **Concentric action for quadriceps (PT; N·m^-1^)** | | | | |  |
| Q Con 60 KL | 214.21 ± 42.48 | 206.95 ± 42.62 | 0.099 | <0.2 - trivial | - |
| Q Con 180 KL | 186.45 ± 28.16 | 186.15 ± 30.37 | 0.424 | <0.2 - trivial | - |
| Q Con 60 SL | 197.51± 25.45 | 196.46 ± 27.2 | 0.505 | <0.2 - trivial | - |
| Q Con 180 SL | 169.05 ± 21.85 | 177.45 ± 24.39 | 0.248 | 0.32 - small | 4.8%; from 0.4% to 9.3 %, (likely beneficial [77, 22, 0]) |
| **Hamstring-to-quadriceps ratios** | | | | |  |
| H/QFUNC 60 KL | 0.76 ± 0.09 | 0.73 ± 0.12 | 0.308 | >-0.2 - trivial | - |
| H/QFUNC 180 KL | 1.04 ± 0.09 | 1.02 ± 0.14 | 0.937 | >-0.2 - trivial | - |
| H/QFUNC 60 SL | 0.72 ± 0.07 | 0.74 ± 0.08 | 0.722 | <0.2 - trivial | - |
| H/QFUNC 180 SL | 1.07 ± 0.12 | 1.03 ± 0.14 | 0.285 | <0.2 - trivial | - |
| H/QCONV 60 KL | 0.70 ± 0.10 | 0.73 ± 0.10 | 0.209 | <0.2 - trivial | - |
| H/QCONV 180 KL | 0.95 ± 0.17 | 0.91 ± 0.14 | 0.308 | >-0.2 - trivial | - |
| H/QCONV 60 SL | 0.67 ± 0.09 | 0.63 ± 0.08 | 0.091 | -0.45 - small | -6.2%; from -12.1% to 0.2%, (likely harmful [82, 16, 2]) |
| H/QCONV 180 SL | 0.93 ± 0.15 | 0.87 ± 0.14 | 0.328 | >-0.2 - trivial | - |

M – mean; SD – standard deviation; Q – quadriceps; H – hamstrings; Con – concentric action; Ecc – eccentric action; KL – Kicking lower limb; SL – stance non-dominant lower limb; H/QFUNC – isokinetic hamstrings eccentric-to-quadriceps concentric functional ratio; H/QCONV – isokinetic hamstrings concentric-to-quadriceps concentric conventional ratio 60, 180 – angular velocities in degrees ∙ s^-1^; *P < 0.05. ^a^ Magnitude thresholds (for change in means divided by baseline SD): <0.20, trivial; 0.20-0.59, small; 0.60-1.19, moderate.

**Table S2.** Descriptive statistics for muscle activation for kicking leg pre and post SAFT90, and magnitude-based inferences for the percent changes of the means in U16.

| **Parameter (Hz).** | **Pre-test (n=11)  Mean ± *SD*** | **Post-test (n=11) Mean ± *SD*** | ***p*** | **Standardized**  **effect**  **size** | **Effect^a^**  **Change in mean; ±90%CI** |
| --- | --- | --- | --- | --- | --- |
| **Thigh muscles** | | | | |  |
| VM Con 60 Ex | 0.99 ± 0.73 | 0.81 ± 0.34 | 0.480 | <0.2 - trivial | - |
| VM Con 180 Ex | 0.92 ± 0.70 | 0.93 ± 0.67 | 0.480 | <0.2 - trivial | - |
| VL Con 60 Ex | 0.79 ± 0.42 | 0.68 ± 0.31 | 0.117 | unclear | - |
| VL Con 180 Ex | 0.87 ± 0.47 | 0.79 ± 0.39 | 0.347 | <0.2 - trivial | - |
| BF Con 60 Fl | 0.67 ± 0.15 | 1.33 ± 1.49 | 0.012* | 2.0 - very large | 57.7%; from 9.7% to 126.7%, (very likely beneficial [97, 2, 2]) |
| BF Con 180 Fl | 0.69 ± 0.27 | 0.72 ± 0.32 | 0.875 | <0.2 - trivial | - |
| BF Ecc 60 Fl | 0.80 ± 0.41 | 0.82 ± 0.31 | 0.875 | <0.2 - trivial | - |
| BF Ecc 180 Fl | 0.80 ± 0.37 | 0.76 ± 0.56 | 0.433 | <0.2 - trivial | - |
| ST Con 60 Fl | 0.87 ± 0.98 | 1.51 ± 2.55 | 0.272 | 0.4 - small | 46.8%; from -7.9% to 134.1%, (likely beneficial [76, 22, 2]) |
| ST Con 180 Fl | 0.55 ± 0.27 | 30.71 ± 0.35 | 0.272 | 0.5 - small | 34.3%; from -7.1% to 94.3%, (likely beneficial [80, 17, 4]) |
| ST Ecc 60 Fl | 1.00 ± 0.64 | 0.82 ± 0.33 | 0.433 | unclear | - |
| ST Ecc 180 Fl | 0.71 ± 0.37 | 1.02 ± 0.86 | 0.272 | unclear | - |
| **Calf muscles** | | | | |  |
| MG Con 60 Fl | 0.94 ± 0.31 | 0.87 ± 0.56 | 0.182 | -0.5 - small | -15.6%; from -41.3% to 21.1%, (possibly harmful [13, 19, 68]) |
| MG Con 180 Fl | 0.88 ± 0.45 | 0.96 ± 1.13 | 0.239 | unclear | - |
| MG Ecc 60 Fl | 0.91 ± 0.30 | 0.76 ± 0.15 | 0.182 | -0.4 - small | -14.2%; from -30.4% to 5.8%, (possibly harmful [4, 22, 74]) |
| MG Ecc 180 Fl | 0.79 ± 0.20 | 0.74 ± 0.24 | 0.308 | <0.2 - trivial | - |
| LG Con 60 Fl | 0.92 ± 0.59 | 0.80 ± 1.00 | 0.117 | unclear | - |
| LG Con 180 Fl | 0.54 ± 0.21 | 0.77 ± 0.72 | 0.433 | unclear | - |
| LG Ecc 60 Fl | 0.77 ± 0.23 | 0.60 ± 0.19 | 0.019* | -0.71 - moderate | -22.2%; from -34.3% to -7.8%, (very likely harmful [0, 4, 96]) |
| LG Ecc 180 Fl | 0.68 ± 0.26 | 0.67 ± 0.21 | 0.937 | <0.2 - trivial | - |

M – mean; SD – standard deviation; Con – concentric action; Ecc – eccentric action; Ex – extension; Fl – flexion; ALS – absolute leg stiffness; RLS – relative leg stiffness; RSI – reactive strength index; VL – vastus lateralis; VM – vastus medialis. BF – biceps femoris; ST – semitendinosus muscle; MG – medial gastrocnemius; LG – lateral gastrocnemius; 60, 180 – angular velocities *P < 0.05.

^a^ Magnitude thresholds (for change in means divided by baseline SD): <0.20, trivial; 0.20-0.59, small; 0.60-1.19, moderate.

**Table S3.** Descriptive statistics (Mean ± SD) for Leg stiffness and RSI parameters pre and post SAFT^90^, and magnitude-based inferences for the changes of the means in U16.

| **Parameter** | **Pre SAFT^90^** | Post SAFT^90^ | Effect | |
| --- | --- | --- | --- | --- |
|  |  |  | Standardized  effect  size | change in mean; ±90%CI |
| Leg stiffness ABS (kN·m^-1^) | 28.0 ± 5.3 | 25.4 ± 4.9 | -0.47 – small* | -9.2%; from -13.8% to 4.5% (very likely harmful [0, 4, 96]) |
| Leg stiffness REL | 35.5 ± 6.7 | 32.1 ± 5.3 | -0.51 – small* | -9.2%; from -13.8% to 4.5% (very likely harmful [0, 3, 97]) |
| RSI | 0.40 ± 0.09 | 0.43 ± 0.07 | 0.38 – small* | 8.0%; from 11.3% to 3.3% (very likely beneficial [97, 3, 0]) |

ABS – absolute leg stiffness, REL - absolute leg stiffness multiplied by body mass and leg length index (value about 0.8). ^a^Magnitude thresholds (for change in means divided by baseline SD): <0.20, trivial; 0.20-0.59, small.

Asterisks indicate effects clear at the 5% level and likelihood that the true effect is substantial as *very likely. All effects are also clear at 0.5% level.

**Table S4.** Descriptive statistics for isokinetic parameters pre and post SAFT^90^, and magnitude-based inferences for the percent changes of the means in U17.

| **Parameter (N·m^-1^)** | **Pre-test (n=11)  Mean ± *SD*** | **Post-test (n=11)  Mean ± *SD*** | ***p*** | **Standardized**  **effect**  **size** | **Effect**  **Change in mean; ±90%CI** |
| --- | --- | --- | --- | --- | --- |
| **Concentric and eccentric action for hamstrings ; N·m^-1^** | | | | |  |
| H Con 60 KL | 159.86 ± 28.12 | 155.75 ± 19.04 | 0.286 | >-0.2 - trivial | - |
| H Con 180 KL | 182.78 ± 23.29 | 176.53 ± 15.28 | 0.182 | -0.23 - small | -3.1%; from -7.6% to 1.7 %, (possibly harmful [3, 42, 55]) |
| H Con 60 SL | 149.71 ± 25.49 | 142.81 ± 29.36 | 0.213 | -0.28 - small | -5.1%; from -11.7.1% to 2.0 %, (possibly harmful [2, 33, 65]) |
| H Con 180 SL | 167.7 ± 19.74 | 162.77 ± 20.02 | 0.221 | -0.24 - small | -3.0%; from -6.7% to -0.8 %, (possibly harmful [1, 39, 60]) |
| H Ecc 60 KL | 175.61 ± 31 | 147.47 ± 18.13 | 0.003** | -0.89 - moderate | -15.4%; from -19.6% to -11.1 %, ( most likely harmful [0, 0, 100]) |
| H Ecc 180 KL | 200.32 ± 17.89 | 191.24 ± 24.7 | 0.075 | -0.54 - small | -4.9%; from -8.1% to -1.5 %, (likely harmful [0, 6, 94]) |
| H Ecc 60 SL | 153.62 ± 26.59 | 142.6 ± 24.96 | 0.012* | -0.40 - small | -7.1%; from -11.1% to -3 %, (likely harmful [0, 7, 92]) |
| H Ecc 180 SL | 195.05 ± 19.72 | 192.35 ± 20.19 | 0.286 | >-0.2 - trivial | - |
| **Concentric action for quadriceps** | | | | |  |
| Q Con 60 KL | 238.65 ± 44.88 | 217.87 ± 39.61 | 0.007** | -0.45 - small | -8.6%; from -12.5% to -4.5 %, (very likely harmful [0, 3, 97]) |
| Q Con 180 KL | 204.55 ± 28.91 | 197.43 ± 30.1 | 0.091 | -0.24 - small | -3.7%; from -12.2% to -3.2%, (possibly harmful [0, 37, 62]) |
| Q Con 60 SL | 226.85 ± 36.56 | 210.77 ± 45.85 | 0.033* | -0.50 - small | -7.8%; from -12.5% to -4.5 %, (likely harmful [0, 5, 95]) |
| Q Con 180 SL | 195.83 ± 29.1 | 189.36 ± 25.3 | 0.032 | -0.20- small | -3.1%; from -5.7% to -0.4%, (possibly harmful [0, 54, 46]) |
| **Hamstring-to-quadriceps ratios** | | | | |  |
| H/QFUNC 60 KL | 0.74 ± 0.09 | 0.69 ± 0.08 | 0.026 | >-0.2 - trivial | - |
| H/QFUNC 180 KL | 0.99 ± 0.12 | 0.98 ± 0.14 | 0.859 | >-0.2 - trivial | - |
| H/QFUNC 60 SL | 0.68 ± 0.09 | 0.69 ± 0.1 | 0.790 | <0.2 - trivial | - |
| H/QFUNC 180 SL | 1.01 ± 0.16 | 1.03 ± 0.14 | 0.374 | <0.2 - trivial | - |
| H/QCONV 60 KL | 0.68 ± 0.12 | 0.73 ± 0.12 | 0.041* | 0.36 - small | 7.3%; from 0.4% to 14.7%, (likely harmful [82, 16, 2]) |
| H/QCONV 180 KL | 0.91 ± 0.17 | 0.91 ± 0.16 | 0.722 | <0.2 - trivial | - |
| H/QCONV 60 SL | 0.67 ± 0.13 | 0.69 ± 0.12 | 0.657 | <0.2 - trivial | - |
| H/QCONV 180 SL | 0.88 ± 0.19 | 0.87 ± 0.16 | 0.859 | >-0.2 - trivial | - |

M – mean; SD – standard deviation; Q – quadriceps; H – hamstrings; Con – concentric action; Ecc – eccentric action; KL – Kicking leg; SL – stance non-dominant leg; H/QFUNC – isokinetic hamstrings eccentric-to-quadriceps concentric functional ratio; H/QCONV – isokinetic hamstrings concentric-to-quadriceps concentric conventional ratio 60, 180 – angular velocities; *P < 0.05. ^a^ Magnitude thresholds (for change in means divided by baseline SD): <0.20, trivial; 0.20-0.59, small; 0.60-1.19, moderate. Effects in bold are also clear at 0.5% level.

**Table S5.** Descriptive statistics for muscle activation for kicking leg pre and post SAFT^90^, and magnitude-based inferences for the percent changes of the means in U17.

| **Parameter (Hz)** | **Pre-test (n=11)  Mean ± *SD*** | **Post-test (n=11) Mean ± *SD*** | ***p*** | **Standardized**  **effect**  **size** | **Effect^a^**  **Change in mean; ±90%CI** |
| --- | --- | --- | --- | --- | --- |
| **Thigh muscles** | | | | |  |
| VM Con 60 Ex | 0.45 ± 0.24 | 0.46 ± 0.39 | 0.950 | unclear | - |
| VM Con 180 Ex | 0.36 ± 0.17 | 0.30 ± 0.05 | 0.953 | >-0.2 - trivial | - |
| VL Con 60 Ex | 0.45 ± 0.18 | 0.31 ± 0.06 | 0.441 | -0.71 - moderate | -26.9%; from -42.4% to -7.1%, ( likely harmful [1, 5, 94]) |
| VL Con 180 Ex | 0.45 ± 0.18 | 0.28 ± 0.05 | 0.015* | -0.99 - moderate | -35.4%; from -44.8% to -11.4%, (very likely harmful [0, 2, 98]) |
| BF Con 60 Fl | 0.61 ± 0.30 | 0.43 ± 0.18 | 0.260 | >-0.2 - trivial | - |
| BF Con 180 Fl | 0.47 ± 0.28 | 0.37 ± 0.11 | 0.374 | >-0.2 - trivial | - |
| BF Ecc 60 Fl | 0.43 ± 0.24 | 0.41 ± 0.22 | 0.594 | >-0.2 - trivial | - |
| BF Ecc 180 Fl | 0.55 ± 0.49 | 0.38 ± 0.15 | 0.441 | >-0.2 - trivial | - |
| ST Con 60 Fl | 0.53 ± 0.14 | 0.40 ± 0.11 | 0.038* | -0.92 - moderate | -25.3%; from -47.3% to -6.0%, (very likely harmful [1, 4, 95]) |
| ST Con 180 Fl | 0.41 ± 0.21 | 0.36 ± 0.14 | 0.767 | >-0.2 - trivial | - |
| ST Ecc 60 Fl | 0.54 ± 0.20 | 0.37 ± 0.11 | 0.028* | -0.91 - moderate | -30.0%; from -51,6% to 1.0%, (very likely harmful [0, 3, 97]) |
| ST Ecc 180 Fl | 0.47 ± 0.30 | 0.39 ± 0.12 | 0.515 | unclear | - |
| **Calf muscles** | | | | |  |
| MG Con 60 Fl | 0.83 ± 0.29 | 0.72 ± 0.28 | 0.182 | >-0.2 - trivial | - |
| MG Con 180 Fl | 0.75 ± 0.42 | 0.83 ± 0.40 | 0.239 | unclear | - |
| MG Ecc 60 Fl | 0.76 ± 0.30 | 0.59 ± 0.09 | 0.182 | -0.50 - small | -18.2%; from -32.4% to -1.1%, (likely harmful [1, 12, 86]) |
| MG Ecc 180 Fl | 0.83 ± 0.51 | 0.52 ± 0.15 | 0.308 | -0.66 - moderate | -31.6%; from -49.4% to -7.5%, (likely harmful [1, 6, 93]) |
| LG Con 60 Fl | 0.62 ± 0.37 | 0.51 ± 0.31 | 0.117 | unclear | - |
| LG Con 180 Fl | 0.47 ± 0.26 | 0.35 ± 0.21 | 0.433 | unclear | - |
| LG Ecc 60 Fl | 0.61 ± 0.33 | 0.43 ± 0.15 | 0.019* | -0.66 - moderate | -29.9%; from -46.4% to -8.5%, (likely harmful [1, 6, 94]) |
| LG Evc 180 Fl | 0.58 ± 0.19 | 0.37 ± 0.12 | 0.937 | -1.40 - large | -37.1%; from -45.6% to -27.2%, (most likely harmful [0, 0, 100]) |

M – mean; SD – standard deviation; Con – concentric action; Ecc – eccentric action; Ex – extension; Fl – flexion; ALS – absolute leg stiffness; RLS – relative leg stiffness; RSI – reactive strength index; VL – vastus lateralis; VM – vastus medialis. BF – biceps femoris; ST – semitendinosus muscle; MG – medial gastrocnemius; LG – lateral gastrocnemius; 60, 180 – angular velocities *P < 0.05.

a Magnitude thresholds (for change in means divided by baseline SD): <0.20, trivial; 0.20-0.59, small; 0.60-1.19, moderate. Effects in bold are also clear at 0.5% level.

**Table S6.** Descriptive statistics (Mean ± SD) for Leg stiffness and RSI parameters pre and post SAFT90, and magnitude-based inferences for the changes of the means in U17.

|  | **Pre SAFT^90^** | **Post SAFT^90^** | Standardized  effect  size | Effect  change in mean; ±90%CI |
| --- | --- | --- | --- | --- |
| Leg stiffness ABS (kN·m^-1^) | 30.7 ± 7.1 | 27.2 ± 4.6 | -0.43 – small* | -10.2%; from -14.8% to -5.3% (very likely harmful [0, 4, 96]) |
| Leg stiffness REL | 37.1 ± 8.3 | 33.1 ± 6.3 | -0.44 – small* | -10.2%; from -14.8% to -5.3% (very likely harmful [0, 4, 96]) |
| RSI | 0.37± 0.04 | 0.38 ± 0.05 | 0.24 – small* | 2.5%; from -2.2% to 7.4% (possibly beneficial [56,38, 5]) |

ABS – absolute leg stiffness, REL - absolute leg stiffness multiplied by body mass and leg length index (value about 0.8).

a Magnitude thresholds (for change in means divided by baseline SD): <0.20, trivial; 0.20-0.59, small.

Asterisks indicate effects clear at the 5% level and likelihood that the true effect is substantial as *very likely. Effects in bold are also clear at 0.5% level.
